# Prediction model of axillary lymph node status using automated breast ultrasound (ABUS) and ki-67 status in early-stage breast cancer

**DOI:** 10.1186/s12885-022-10034-3

**Published:** 2022-08-28

**Authors:** Qiucheng Wang, Bo Li, Zhao Liu, Haitao Shang, Hui Jing, Hua Shao, Kexin Chen, Xiaoshuan Liang, Wen Cheng

**Affiliations:** 1 Department of Ultrasound, Harbin Medical University Cancer Hospital, No. 150, Haping Road, Nangang District, Harbin, Heilongjiang Province China; 2 Department of Pathology, Harbin Medical University Cancer Hospital, No. 150, Haping Road, Nangang District, Harbin, Heilongjiang Province China; 3 Department of Breast Surgery, Harbin Medical University Cancer Hospital, No. 150, Haping Road, Nangang District, Harbin, Heilongjiang Province China; 4 Department of Interventional Ultrasound, Harbin Medical University Cancer Hospital, Harbin, 150081 Heilongjiang China

**Keywords:** Automated breast ultrasound, Early-stage breast cancer, Axillary lymph node metastasis, Ki-67, Retraction phenomenon

## Abstract

**Background:**

Automated breast ultrasound (ABUS) is a useful choice in breast disease diagnosis. The axillary lymph node (ALN) status is crucial for predicting the clinical classification and deciding on the treatment of early-stage breast cancer (EBC) and could be the primary indicator of locoregional recurrence. We aimed to establish a prediction model using ABUS features of primary breast cancer to predict ALN status.

**Methods:**

A total of 469 lesions were divided into the axillary lymph node metastasis (ALNM) group and the no ALNM (NALNM) group. Univariate analysis and multivariate analysis were used to analyze the difference of clinical factors and ABUS features between the two groups, and a predictive model of ALNM was established. Pathological results were as the gold standard.

**Results:**

Ki-67, maximum diameter (MD), posterior feature shadowing or enhancement and hyperechoic halo were significant risk factors for ALNM in multivariate logistic regression analysis (*P* < 0.05). The four risk factors were used to build the predictive model, and it achieved an area under the receiver operating characteristic (ROC) curve (AUC) of 0.791 (95% CI: 0.751, 0.831). The accuracy, sensitivity and specificity of the prediction model were 72.5%, 69.1% and 75.26%. The positive predictive value (PPV) and negative predictive value (NPV) were 66.08% and 79.93%, respectively. Distance to skin, MD, margin, shape, internal echo pattern, orientation, posterior features, and hyperechoic halo showed significant differences between stage I and stage II (*P* < 0.001).

**Conclusion:**

ABUS features and Ki-67 can meaningfully predict ALNM in EBC and the prediction model may facilitate a more effective therapeutic schedule.

**Supplementary Information:**

The online version contains supplementary material available at 10.1186/s12885-022-10034-3.

## Background

The axillary lymph node (ALN) status is crucial for predicting the clinical classification and decisions on treatment of early-stage breast cancer (EBC) and could be the primary indicator of locoregional recurrence [1, 2]. Lymph node dissection can cause lymphedema, which can further contribute to pain, bloating, pressure, fatigue, and functional restriction [3, 4]. To reduce the occurrence of lymphedema, sentinel lymph node biopsy (SLNB) is mainly used before surgery. SLNB is the main technology used to assess axillary lymph node metastasis (ALNM) status in patients with breast cancer and imaging-negative ALNs because of less physical injury than surgical dissection. However, it may also result in some complications such as wound infection, hematoma, abnormal sensation, local tension, functional restriction, lymphedema, and high financial burden [1, 5]. Conventional handheld ultrasound is widely used in predicting ALN status according to focal changes in the cortical morphologic features of ALN [6]. However, radiologists often cannot find any signs of metastasis on US images of clinically negative lymph nodes. Variable techniques and different criteria for malignant ALNs can result in unnecessary biopsy or false negative results [7]. In addition, early ALNM often does not cause changes in structure or size on ultrasound [8]. Therefore, some researchers reported that breast ultrasound features could help provide some information or likelihood of ALNM [9, 10].

Automated breast ultrasound (ABUS), as a noninvasive and effective imaging modality, has been increasingly widely used on account of its automated volumetric scanning of the breast lesions with high frequency broadband transducers [11, 12]. ABUS can reconstruct three-dimensional (3D) images of the breast lesion volume, including coronal, axial, and sagittal views, in which the coronal view has been shown to improve early detection in dense breasts and diagnostic accuracy because of the “retraction phenomenon”, which was described as a convergence sign from the surface of the solid nodule with hyperechoic straight lines radiating perpendicularly [13–15]. However, according to our investigation, no study has used the ABUS features of primary breast cancer to predict ALNM status. In addition, it has been reported that tumor clinicopathologic characteristics, such as Ki-67 expression status and molecular subtype (MS), that is, lumina A, lumina B, HER-2 overexpression, triple negative subtype, might be associated with ALNM [9, 16, 17]. In summary, the purpose of this study was to investigate the correlation among ABUS features, MS, clinical factors of EBC lesions and ALNM to build a useful prediction model of ALNM in EBC.

## Methods

The retrospective study was approved by the Institutional Review Board of Harbin medical university cancer hospital. The need for informed consent was waived because of the retrospective nature of the cohort study.

### Patients

**Fig. 1:**
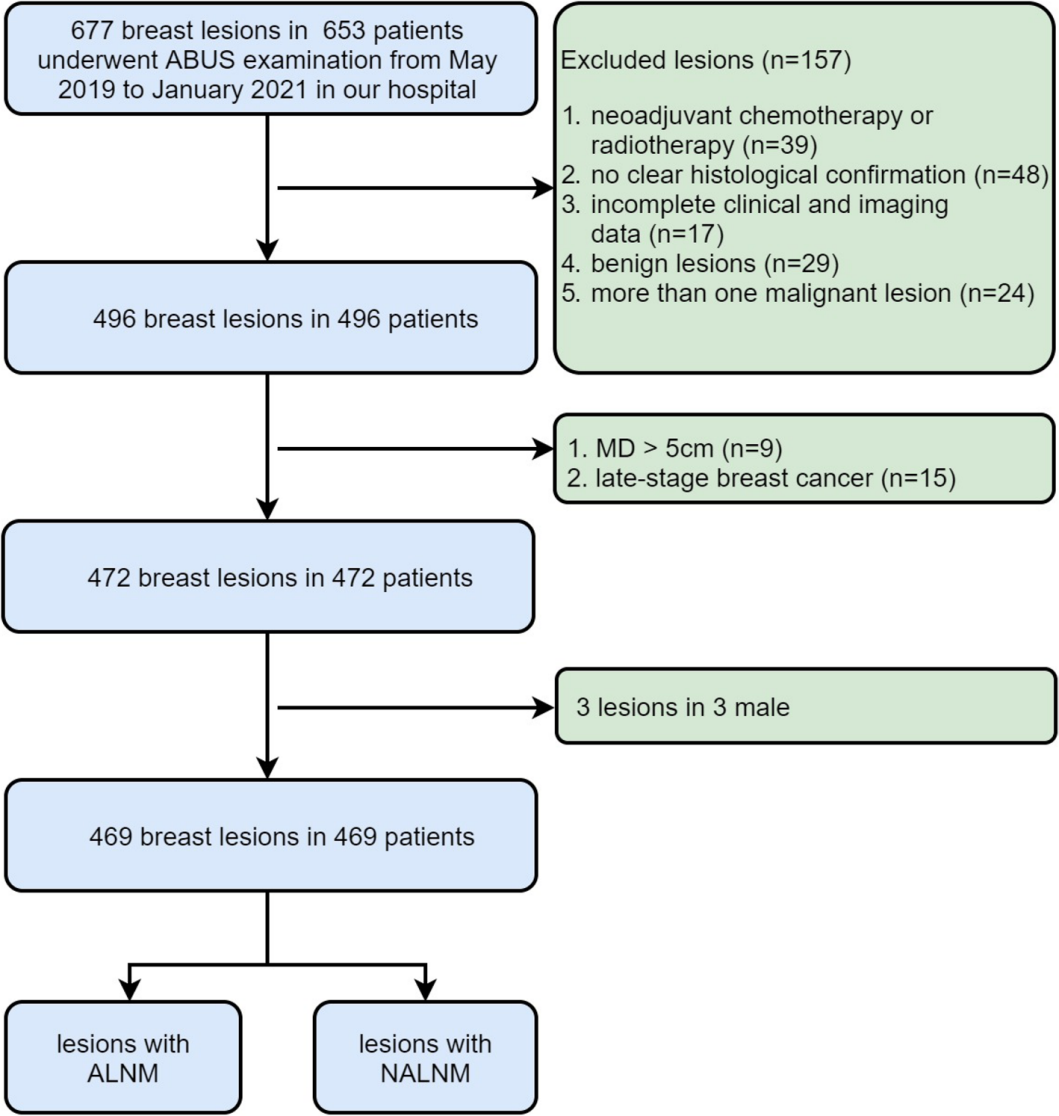
Flowchart of procedures in the breast lesion selection. ABUS = automated breast ultrasound. ALNM = axillary lymph node metastasis, NALNM = no axillary lymph node metastasis, MD = maximum diameter

Patients with breast cancer lesions, diagnosed by surgery or biopsy specimen, between May 2019 and January 2021 were included in this study. All patients consecutively underwent ABUS before surgery or biopsy. Clinical information of patients was recorded, and ABUS examinations were performed by skilled technologists. The patient collection process is shown in Fig. 1. Mastectomy, breast conserving surgery specimens were examined for estrogen-receptor (ER), progesterone-receptor (PR), human epidermal growth factor receptor 2 (HER-2), Ki-67 and P53. Each clinical factor, including age, marital status, fertility status, menopause, BMI, Ki-67, P53, ER, PR, HER-2 and MS was recorded. The inclusion criteria for the patients were as follows: (1) only one breast lesion in each patient was confirmed as breast cancer by pathology, (2) breast cancer with 0.1 cm < MD ≤ 5 cm and in cancer stage I and II proved without distant metastasis by bone scan, liver sonography, chest CT scan or PET-CT, (3) no neoadjuvant chemotherapy or radiotherapy before ABUS examination, (4) ALN status was clearly confirmed by SLNB or ALN dissection (ALND), (5) ABUS features of EBC could be clearly showed and with high quality images, and (6) complete data and clinical information. The exclusion criteria were as follows: (1) neoadjuvant chemotherapy or radiotherapy, (2) no clear histological confirmation, (3) incomplete clinical and imaging data, (4) benign lesions, (5) more than one malignant lesion, (6) MD > 5 cm (7) late-stage breast cancer, and (8) male patients.

### ABUS examination

The Invenia™ Automated Breast Ultrasound System (Invenia ABUS, Automated Breast Ultrasound System, GE Healthcare, Sunnyvale, CA, USA) with an automated 6–14 MHz linear broadband transducer (covering volumes of 15.4 × 17.0 × 5.0 cm) was used in the study. The patients were placed similar to those with conventional US. Thus, anteroposterior, medial, and lateral orientation items of volume data were obtained, and, if required, superior and inferior orientations were performed additionally. After acquisition, all the images were sent to the workstation for review by the two experienced radiologists (with 5 years and 4 years of work experience in ABUS). ABUS imaging analysis is based on the same features as conventional ultrasound (CUS), simultaneously adding 3D analysis by generating a coronal view [13, 18, 19]. The imaging features (axial, sagittal, and coronal view) included MD, location (upper outer quadrant, outer lower quadrant, upper inner quadrant, inner lower quadrant), shape (regular, irregular), margin (circumscribe, spiculated, angular, indistinct), orientation (parallel, nonparallel), echo pattern (hypoechoic, heterogeneous/complex cystic and solid, hyperechoic), posterior features (no posterior features, shadowing, enhancement, combined pattern), calcifications (no, micro, macro), hyperechoic halo (negative, positive), and “retraction phenomenon” in the coronal view (negative, positive) [19, 20]. Unfortunately, due to the limitations of the examination process, ABUS cannot assess the status of ALNs.

### Histopathological analysis

The ER, PR, Ki-67 and P53 statuses of all patients were determined by immunohistochemical analysis, and HER-2 were determined by immunohistochemistry or fluorescent in situ hybridization (FISH). The definitions of P53, ER, PR, HER-2 status was as follows: Ki-67 status (negative < 14%, positive ≥ 14%), P53 status (negative < 10%, positive ≥ 10%), ER and PR status (negative ≤ 1%, positive 1 > %), and HER-2 status (negative 0 or 1 + , positive 3 + , borderline 2 +). FISH was performed to make a final determination when HER-2 status was 2 + . If the gene-to-chromosome ratio was more than 2.0, HER-2 was considered gene amplification [21–23]. The patients were categorized into four MSs based upon previously validated clinicopathological criteria [23]. The axillary lymph node status was recorded.

### Data analysis

According to the pathological results, all lesions were divided into the ALNM group and NALNM group. The ABUS features and each clinical factor between the two groups were compared by univariate analysis. The correlation between each variable and ALNM was studied by univariate analysis and multivariate analysis. According to the obtained significant results, a prediction model of ALNM was established. The specificity and sensitivity of the significant variables were drawn in ROC space, and AUCs with 95% confidence intervals (CIs) were calculated. In addition, the correlation between ABUS features and cancer staging [24] was studied. Furthermore, the correlation among MD, ABUS features and Ki-67 was also analyzed in the same way. All cases underwent cancer staging. The correlation between ABUS features and cancer staging were analyzed.

### Statistical analysis

SAS9.4 software was used for statistical analysis. The mean ± standard deviation was used to describe measurement data conforming to the normal distribution. T test was used for comparison between groups. If data did not conform to the normal distribution, the median and quartile (q1, q3) were used for statistical description. Rank sum test were used for comparison between the groups. The count data were described by count and percentage, and the Chi-square test was used for comparisons between the groups. Univariate logistic regression analysis was performed and covariates with a *P* < 0.05 was considered significant (to avoid eliminating significant variables). The variables found to be significant in the univariate analysis were included in the multivariate analysis. Logistic regression was used for ALNM prediction model development with the variables identified as significant in the univariate analysis (*P* < 0.05). The Youden Index was used to select the cutoff value of the predicted probability. *P* < 0.05 indicates that the differences were statistically significant.

## Results

In total, there were 469 female patients in the study with 469 EBC lesions. The average age of the patients was 53.2 ± 10.05 years (range, 27–79 years) and mean lesion MD was 2.276 ± 0.90 cm (range, 0.6–5.0 cm) as measured by ABUS. Among these lesions, 291 (62.05%, 291/469) had NALNM, and 178 (37.95%, 178/469) had ALNM. A total of 165 lesions (35.18%, 165/469) were classified as stage I, and 304 (64.82%, 304/469) were classified as stage II. Body Mass Index (BMI) of all the patients was 23.717 ± 3.107. The comparison of clinical factors and ABUS features between EBC lesions with and without ALNM is shown in Tables 1 and 2. The correlation between ABUS features and cancer staging was analyzed, and the results are shown in Table 2. Ki-67, HER-2, MS, MD, shape, echo pattern, calcifications, posterior features and hyperechoic halo were significantly different between the ALNM and NALNM groups (*P* < 0.05). Lesions with ALNM were significantly larger than those with NALNM [2.587 ± 0.852 cm vs. 2.086 ± 0.893 cm] (*P* < 0.001). Lesions with shorter distances to the skin [0.628 ± 0.462 vs. 0.747 ± 0.591] and larger MDs [2.587 ± 0.852 cm vs. 2.086 ± 0.893 cm] were more prone to occur in stage II (*P* < 0.001).

**Table 1:** Comparison of clinical factors between ALNM^*^ and NALNM in EBC lesions

Variables	Total (*n* = 469)	NALNM (%) (*n* = 291)	ALNM (%) (*n* = 178)	*P*
Age				0.651
< 40	34	24(70.6)	10(29.4)	
40 ~ 49	156	100(64.1)	56(35.9)	
50 ~ 59	152	94(61.8)	58(38.2)	
60 ~ 69	99	57(57.6)	42(42.4)	
≥ 70	28	16(57.1)	12(42.9)	
Marital status				0.869
Unmarried	3	2(66.7)	1(33.3)	
Married	466	289(62.0)	177(38.0)	
Fertility status				0.893
No	10	6(60.0)	4(40.0)	
Yes	459	285(62.1)	174(37.9)	
Menopause				0.247
No	240	155(64.6)	85(35.4)	
Yes	229	136(59.4)	93(40.6)	
BMI	23.717 ± 3.107	23.507 ± 2.905	24.060 ± 3.384	0.061
Smoke				0.788
No	462	287(62.1)	175(37.9)	
Yes	7	4(57.1)	3(42.9)	
Histologic type				0.239
IDC	443	273(61.6)	170(38.4)	
Lobular	17	10(58.8)	7(41.2)	
Other	9	8(88.9)	1(11.1)	
Ki-67				< 0.0001
Negative	170	146(85.9)	24(14.1)	
Positive	299	145(48.5)	154(51.5)	
P53				0.657
Negative	148	94(63.5)	54(36.5)	
Positive	321	97(61.4)	124(38.6)	
ER				0.051
Negative	87	46(52.9)	41(47.1)	
Positive	382	245(64.1)	137(35.9)	
PR				0.146
Negative	122	69(56.6)	53(43.3)	
Positive	347	222(64.0)	125(36.0)	
HER-2				0.001
Negative	357	237(66.4)	120(33.6)	
Positive	112	54(48.2)	58(51.8)	
MS				< 0.0001
Lumina A	136	119 (87.5)	17(12.5)	
Lumina B	254	128(50.39)	126(49.61)	
Her-2 overexpression	42	24(57.1)	18(42.9)	
TN	37	20(54.1)	17(45.9)	
Location				0.056
Inner lower quadrant	25	19(76.0)	6(24.0)	
Upper outer quadrant	294	169(57.5)	125(42.5)	
Outer lower quadrant	77	52(67.5)	25(32.5)	
Upper inner quadrant	73	51(69.9)	22(30.1)	

^*^ *ALNM* Axillary lymph node metastasis, *NALNM* No axillary lymph node metastasis, *EBC* Early-stage breast cancer, *BMI* Body mass index, *IDC* Invasive ductal carcinoma, *ER* Estrogen-receptor, *PR* Progesterone-receptor, *HER-2* Human epidermal growth factor receptor 2, *MS* Molecular subtype, *TN* Triple negative

**Table 2:** Correlation between ABUS features and ALNM in EBC lesions and correlation between ABUS^*^ features and cancer staging

ABUS features		ALN status			Cancer staging		
	Total (*n* = 469)	NALNM (*n* = 291)	ALNM (*n* = 178)	*P*	Stage I (*n* = 165)	Stage II (*n* = 304)	*P*
Distance to nipple	3.099 ± 1.984	3.155 ± 2.004	3.007 ± 1.953	0.4354	3.257 ± 1.918	3.013 ± 2.017	0.204
Distance to skin	0.670 ± 0.514	0.697 ± 0.543	0.626 ± 0.461	0.145	0.747 ± 0.591	0.628 ± 0.462	0.017
MD	2.276 ± 0.909	2.086 ± 0.893	2.587 ± 0.852	< 0.001	1.474 ± 0.377	2.712 ± 0.809	< 0.001
Margin				0.174			0.016
Circumscribe	51	36(70.6)	15(29.4)		26(51.0)	25(49.0)	
Spiculated	240	155(64.6)	85(35.4)		85(35.4)	155(64.6)	
Angular	78	45(57.7)	33(42.3)		29(37.2)	49(62.8)	
Indistinct	100	55(55.0)	45(45.0)		25(25.0)	75(75.0)	
Shape				0.002			0.036
Regular	28	25(89.3)	3(10.7)		15(53.6)	13(46.4)	
Irregular	441	266(60.3)	175(39.7)		150(34.0)	291(66.0)	
Echo pattern				0.011			0.018
Hypoechoic	434	261(60.1)	173(39.9)		146(33.6)	288(66.4)	
Heterogeneous/complex cystic and solid	22	19(86.4)	3(13.6)		10(45.5)	12(54.5)	
Hyperechoic	13	11(84.6)	2(15.4)		9(69.2)	4(30.8)	
Calcifications				0.028			0.138
No	213	146(68.5)	67(31.5)		85(39.9)	128(60.1)	
Micro	248	140(56.5)	108(43.5)		77(31.0)	171(69.0)	
Macro	8	5(62.5)	3(27.5)		3(37.5)	5(62.5)	
Orientation				0.840			0.046
Parallel	431	268(62.2)	163(37.8)		146(33.9)	285(66.1)	
Nonparallel	38	23(60.5)	15(39.5)		19(50.0)	19(50.0)	
Posterior features				< 0.001			< 0.001
No posterior features	313	221(70.6)	92(29.4)		135(43.1)	178(56.9)	
Shadowing	77	28(36.4)	49(63.6)		13(16.9)	64(83.1)	
Enhancement	74	39(52.7)	35(47.3)		17(23.0)	57(77.0)	
Combined pattern	5	3(60.0)	2(40.0)		0(0)	5(100.0)	
Hyperechoic halo				< 0.001			0.005
Negative	332	225(67.8)	107(32.2)		130(39.2)	202(60.8)	
Positive	137	66(48.2)	71(51.8)		35(25.5)	102(74.5)	
Retraction phenomenon				0.208			0.792
Negative	309	198(64.1)	111(35.9)		110(35.6)	199(64.4)	
Positive	160	93(58.1)	67(41.9)		55(34.4)	105(65.6)	

^*^ABUS Automated breast ultrasound, *EBC* Early-stage breast cancer, *ALN* Axillary lymph node, *ALNM* Axillary lymph node metastasis, *NALNM* No axillary lymph node metastasis, *MD* Maximum diameter

Univariate logistic regression analysis showed that Ki-67 positive, HER-2, MD and the AUBS features (Table 3) were significant independent predictors of ALNM in EBC (*P* < 0.05). Lumina A was a protective factor (*P* < 0.001) against ALNM relative to the triple negative (TN) subtype. Echo pattern with heterogeneous/complex cystic and solid pattern was protective factor for ALNM relative to the hypoechoic pattern (*P* = 0.023) (Table 3).

**Table 3:** Univariate and multivariate analysis of the risk factors in EBC^*^ with ALNM

Variables	Univariate analysis		Multivariate analysis	
	*P*	OR(95%CI)	*P*	OR(95%CI)
Age	0.134	1.150 (0.958, 1.380)		
Histologic type				
IDC	Ref			
Lobular	0.816	1.124(0.420,3.099)		
Other	0.132	0.201(0.025,1.619)		
Ki-67	< 0.0001	6.461 (3.976, 10.522)	0.007	3.568(1.419,8.971)
P53	0.657	1.096(0.732, 1.640)		
ER	0.052	0.627(0.392,1.004)		
PR	0.147	0.733(0.482,1.115)		
HER-2	0.001	2.121(1.379,3.264)	0.100	1.701(0.904,3.202)
MS				
TN	Ref			
Lumina A	< 0.001	0.201(0.091, 0.445)	0.535	0.671(0.190,2.367)
Lumina B	0.679	1.157(0.579, 2.315)	0.516	1.310(0.581,2.953)
Her-2 overexpression	0.783	0.882(0.362, 2.148)	0.241	0.496(0.154,1.601)
Location				
Inner lower quadrant	Ref			
Upper outer quadrant	0.078	2.342(0.909, 6.035)		
Outer lower quadrant	0.426	1.522(0.541, 4.283)		
Upper inner quadrant	0.559	1.366(0.480, 3.885)		
Distance to nipple	0.435	0.963(0.876, 1.059)		
Distance to skin	0.159	0.710(0.441, 1.144)		
MD	< 0.001	1.876(1.504, 2.340)	< 0.0001	1.673(1.289,2.171)
Margin				
Circumscribe	Ref			
Spiculated	0.413	1.316(0.682, 2.541)		
Angular	0.140	1.760(0.830, 3.371)		
Indistinct	0.066	1.964(0.956, 4.033)		
Shape	0.006	5.482(1.631, 18.434)	0.306	2.034(0.522,7.921)
Internal echo pattern				
Hypoechoic	Ref			
Heterogeneous / complex cystic and solid	0.023	0.238(0.069, 0.817)	0.056	0.260(0.065,1.036)
Iso- / hyperechoic	0.095	0.274(0.060, 1.253)	0.623	0.667(0.132,3.355)
Calcifications				
No	Ref	Ref		
Micro	0.008	1.681(1.146, 2.465)	0.381	1.238(0.768,1.995)
Macro	0.719	1.307(0.304, 5.631)	0.860	0.845(0.129,5.525)
Orientation	0.840	1.072(0.544, 2.114)		
Posterior features				
No posterior features	Ref	Ref		
Shadowing	< 0.001	4.204(2.489, 7.100)	< 0.001	4.446(2.395,8.256)
Enhancement	0.004	2.156(1.285, 3.616)	0.019	2.156(1.135,4.098)
Combined pattern	0.609	1.601(0.263, 9.743)	0.986	1.018(0.137,4.098)
Hyperechoic halo	< 0.0001	2.262(1.506, 3.397)	0.004	2.033(1.254,3.294)
Retraction phenomenon in coronal view	0.208	1.285(0.869, 1.899)		

^*^ *EBC* Early-stage breast cancer, *ALNM* Axillary lymph node metastasis, *BMI* Body mass index, *IDC* Invasive ductal carcinoma, *ER* Estrogen-receptor, *PR* Progesterone-receptor, *HER-2* Human epidermal growth factor receptor 2, *MS* Molecular subtype, *TN* Triple negative, *MD* Maximum diameter

In multivariate logistic regression analysis, Ki-67, MD, posterior feature shadowing, posterior feature enhancement and hyperechoic halo were significant predictors of ALNM (*P* < 0.005) (Table 3). Based on the results of multivariate analysis, we established the ALNM prediction model and the equation was as follows:

$$\mathrm Y=-3.544\;+\;1.853\;\times\;\mathrm{Ki}67\;+\;0.499\;\times\;\mathrm{MD}\;+\;\left(1.493\;\times\;\mathrm{shadowing}\;\mathrm{or}\;0.516\;\times\;\mathrm{enhancement}\;\mathrm{or}\;-0.190\;\times\;\mathrm{combined}\;\mathrm{pattern}\right)\;+\;0.718\;\times\;\mathrm{hyperechoic}\;\mathrm{halo}.\;p=e^y/\left(e^y+1\right)$$

The p denotes the probability of ALNM with a cutoff value of 0.4424. The e denotes the natural logarithm with a value of 2.71828. The accuracy, sensitivity and specificity of the model were 72.5%, 69.1% and 75.26%, respectively. The positive predictive value (PPV) and negative predictive value (NPV) were 66.08% and 79.93%, respectively.

**Fig. 2:**
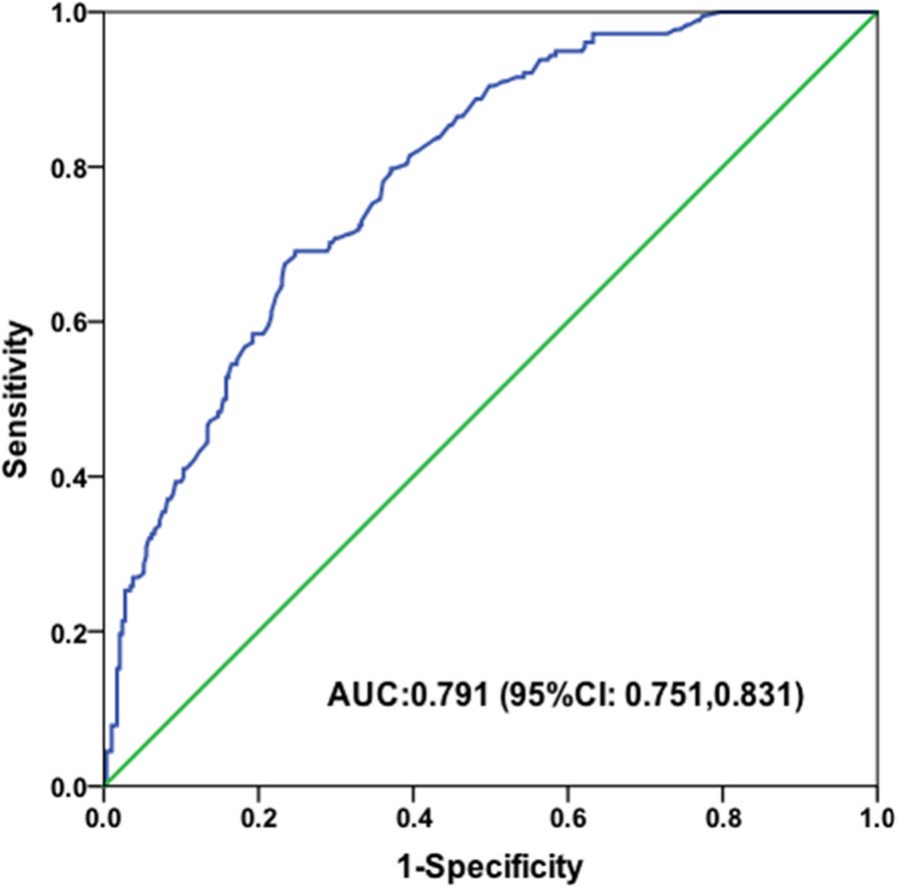
Performance of the model in predicting ALNM of EBC. ALNM = axillary lymph node metastasis, EBC = early-breast cancer, AUC = area under the receiver operating characteristic (ROC) curve

The ROC curve was drawn and AUC was calculated (Fig. 2). It showed moderate predictive efficacy with an AUC of 0.791 (95% CI, 0.751–0.831).

**Fig. 3:**
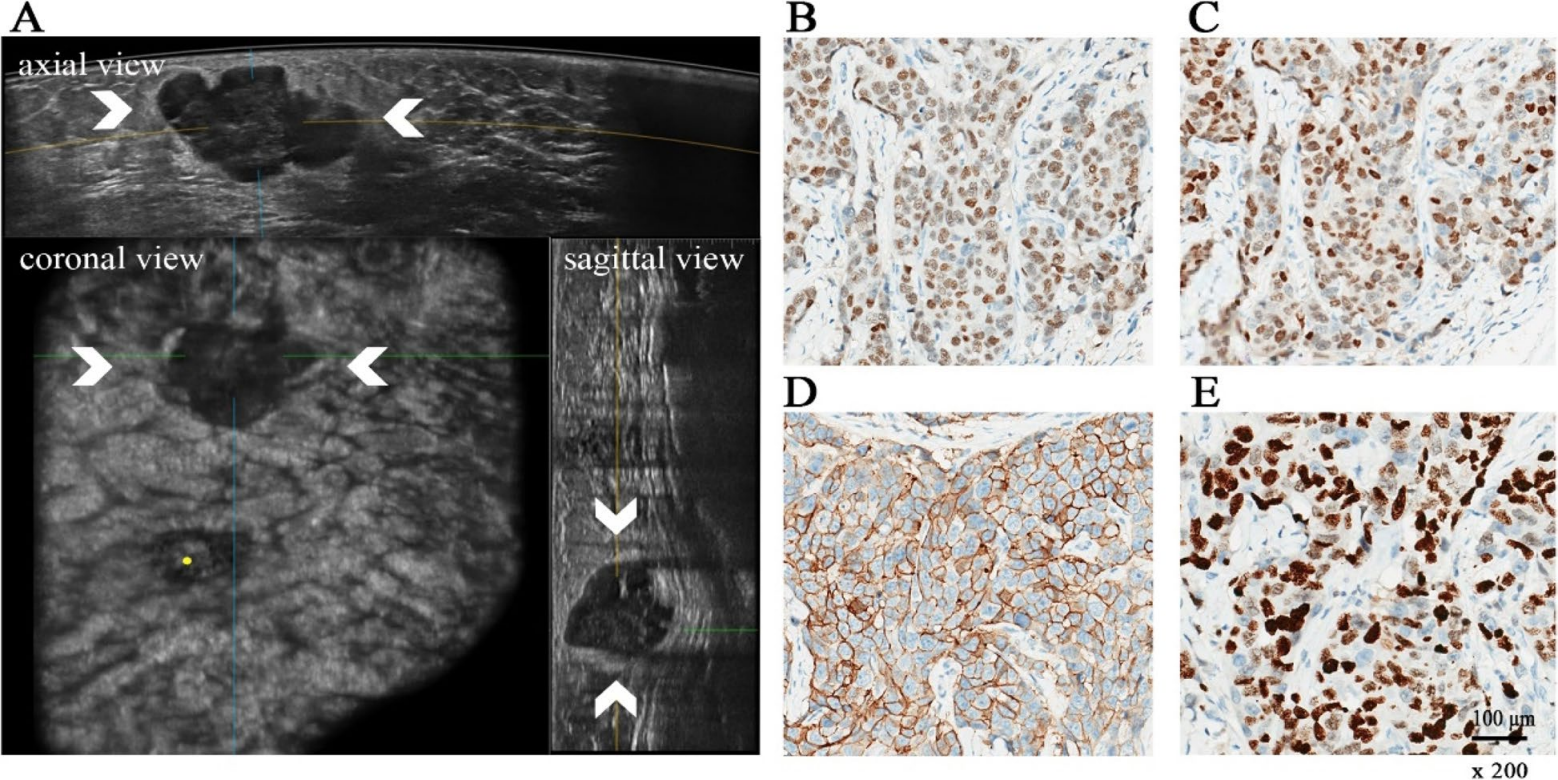
**A** ABUS image of 51-year-old woman in stage II (MD = 3.1 cm) breast cancer with ALNM. The coronal view shows proper nipple position (yellow point). ABUS detected a big hypoechoic lesion (arrows) on outer quadrant in the left breast with irregular shape, lobulated margin, posterior feature enhancement and positive hyperechoic halo. **B, C, D, E** ER ( +), PR ( +), HER-2 (3 +), ki-67 = 80%; (scale bar = 100 μm, × 200). The model predicted that the lesion probably has ALNM (p = 0.75). ABUS = Automated breast ultrasound, MS was Lumina B. MD = maximum diameter, MS = molecular subtype, ALNM = axillary lymph node metastasis. ER = estrogen-receptor, PR = progesterone-receptor, HER-2 = human epidermal growth factor receptor 2

In this study, we further analyzed the correlation among MD, ABUS features and Ki-67 status. The correlation between Ki-67 and MD was statistically significant; that is, the larger the MD of breast cancer lesions was, the higher the value of Ki-67 (Spearman correlation coefficient, r = 0.291, *P* < 0.001). As shown in Table S1, margin, shape, calcifications, posterior features and retraction phenomenon in the coronal view had significant differences in negative and positive Ki-67 status (*P* < 0.05). Lesions with circumscribed/angular or indistinct margins, irregular shape, micro calcifications, combined pattern or negative retraction phenomenon may have higher Ki-67 status. This result may provide helpful information in for the prediction of Ki-67 status. The typical cases are demonstrated in Fig. 3 and Fig. S1.

## Discussion

An accurate evaluation of further diagnosis and ALN status might be beneficial for treatment selection as well as for the assessment of prognosis. In our study, we successfully used ABUS 3D features, MD and Ki-67 status to build a prediction model for predicting ALNM in EBC. Ki-67, MD, posterior feature shadowing, posterior feature enhancement and hyperechoic halo were significant risk factors in predicting ALNM.

Some studies reported that size larger than 2 cm was significantly related to ALNM. Lesions in this study with ALNM were significantly larger than those of NALNM (MD, 2.587 ± 0.852 cm vs. 2.086 ± 0.893 cm, *P* < 0.001). Breast cancer cells can migrate to the ALN via the lymphatic plexuses and network in the breast parenchyma and interstitium. The inconsistent edge of the tumors may promote tumor cell invasion into the adjacent tissues at different growth rates. This can contribute to the increase in tumor size and ALNM [25–27]. The maximum tumor diameter was also significantly associated with high Ki-67 status in this study.

Ki-67 protein expression has been confirmed to be correlated with cell proliferation and the active phases of the cell cycle. Generally, high levels of Ki-67 expression are strongly associated with more proliferation and poor prognosis [28, 29], and are a significant predictor of ALNM [30, 31]. In our investigation, the number of Ki-67 positive cancer in the ALNM group was 154 (51.51%), the proportion of which was the highest (*P* < 0.001). Ki-67 positive was a significant predictor of ALNM in multivariate regression analysis. As reported in previous studies, assessment of the Ki-67 index represents an easy and reliable method for evaluating cell proliferative activity in breast cancer. The rapid proliferation and invasion of tumor cells will cause larger size, irregular shape, uncircumscribed margin, heterogeneous or positive hyperechoic halo in ultrasound features of EBC [30, 31]. Conveniently we can determine the status of Ki-67 by core needle biopsy, we still want to observe the relationship between ki-67 and ABUS features, so that we can make a prediction of the status of ki-67 preliminary. In our study, lesions with circumscribed/angular or indistinct margin, irregular shape, microcalcifications, posterior features, combined pattern or negative retraction phenomenon were prone to have higher Ki-67 status.

Rapid proliferation, high content of collagen fibers in the interstitial tissue and invasion into the adjacent tissues also contribute to the ultrasound features of irregular shape, uncircumscribed margin and shadowing [32]. The posterior shadowing is caused by the increased and disordered arrangement of collagen fibers in the tumor stroma and breast cancer with posterior shadowing is more typically slow growing and low-grade [33]. However, this may allow breast cancer with low proliferative rates sufficient latency period before palpable or symptomatic. A relatively long growth period may lead to a higher chance of ALNM. It has also been previous reported that posterior shadowing was independent risk factor for a heavy axillary nodal tumor burden [34]. Post acoustic enhancement, as a feature of high-grade tumor, is caused by increased cellularity in the mass with prominent large tumor nests and little fibrous stroma [35, 36]. The appearance of the hyperechoic halo is caused by the infiltration of the cancerous tissue into the peripheral fine lymphatic vessels, which is caused by direct infiltration of the cancerous tissue. To a certain extent, it reflects the degree of cancer cell invasion and is an important indicator of poor prognosis [37, 38]. The above conclusion demonstrated our research results from a pathological point of view. In our study, lesions with posterior feature shadowing, enhancement and hyperechoic halo were more likely to have ALNM than those without these features.

A characteristic manifestation of ABUS in the coronal plane is the convergence sign of the “retraction phenomenon”. ABUS has been proven to improve early detection in dense breasts and diagnostic accuracy because of the retraction phenomenon. In addition, the retraction pattern is generally more severe in luminal A than in luminal B, HER-2-enriched and TN IDCs [39]. The retraction phenomenon is caused by a desmoplastic reaction surrounding malignant lesions, which can prevent the rapid invasion and metastasis of breast cancer cells and give the body a time to respond to the tumor. This is the reason why luminal A lesions grow at a slower rate than the other MSs of breast cancer [39, 40]. It was also reported that the masses in the luminal A subtype were prone to the smallest amount of growth [41]. In our study, lumina A was a protective factor for ALNM relative to TN. Lesions with negative retraction phenomena were more prone to have high Ki-67 status. Therefore, we supposed that there was a certain relationship among them. In summary, the “retraction phenomenon” is an effective feature for the diagnosis of breast cancer. However, its appearance may also prevent early ALNM in a sense.

Marital status, pregnancy and fertility status, obesity, menopause status, smoking and alcohol habit have been reported to be correlated with breast cancer. Late menopause, smoking, alcohol use and obesity can increase breast cancer risk [42–44]. Every additional birth can reduce the risk of breast cancer by 10% [45]. However, in our study these factors showed no significant difference in the presence or absence of ALNM in EBC.

The sensitivity of our prediction model was 69.1%, which was higher than the results that the sensitivity value ranged from 26.4% to 75.9% of CUS. The specificity of our study was 75.26%, which was consistent with the results ranged from 55.6% to 97.3% of CUS [8, 9, 46]. The NPV of the prediction model was 79.93%, which was higher than some previse researches [8, 46]. It can help identify more negative lymph nodes, which may help reduce unnecessary core needle biopsy.

Our prediction model showed moderate predictive efficacy with an AUC of 0.791. This result is similar to those of recent studies, which have investigated the potential value of CUS features of breast lesions in predicting ALNM with reported AUCs ranging from 0.731 to 0.848 [8, 47]. There were a lot of studies used CUS features of breast cancer and ALN to evaluate ALNM and indicated that the tumor characteristics were associated with lymph node metastasis [8–10, 47]. Although ABUS cannot assess the status of axillary lymph nodes, it can provide a 3D imaging for breast tumors, which can provide more information about the tumors. Furthermore, the operator-independent automatic scanning system can make the images more standardized, which is more conducive to our accurate interpretation of the images. In addition, our model can assist ABUS in assessing the status of axillary lymph nodes to reduce the disadvantage of not being able to check the axilla. Therefore, on the basis of the present study, we suggested that ABUS might be a better diagnostic method than CUS. We hypothesized that it could be used as predictor in clinically negative ALNs and could omitting SLNB in the future. In order to test the hypothesis, multi-center study and more patients are needed. The present model was still not a substitute for SLNB, and SLNB was still needed to avoid false negatives.

Our study has several limitations: First, due to the retrospective analysis and single-center study, our results may be biased. Second, ABUS cannot observe the Color Doppler information and ALN features, so in this study we did not investigate the relationship among color Doppler flow, ALN features and ALNM. Third, only a few patients had CUS data of tumors, so our model failed to predict ALNS with CUS.

## Conclusions

ABUS has been increasingly widely used in clinical workflows for convenient operation, excellent repeatability and adjunct screening of women with dense breasts.

Although the results of the predictive model established based on ABUS cannot fundamentally change the decision-making of SLNB and the method of surgery of EBC, it can provide more clinical references. In the future, multimode methods, such as ABUS combined with CUS, contrast-enhanced ultrasound, ultrasound elastography evaluation or artificial intelligence, should be necessarily used to improve diagnostic performance.

## Supplementary Information

**Additional file 1:** **Figure S1.** (A) ABUS image of 48-year-old woman in stage II (MD = 2.1 cm) breast cancer with ALNM. The coronal plane shows proper nipple position (yellow point). ABUS detected a big hypoechoic lesion (arrows) on outer quadrant in left breast with irregular shape, angular margin, microcalcification, posterior features shadowing, positive hyperechoic halo and retraction phenomenon. (B, C, D, E) ER (+), PR (+), HER2 (1+), Ki-67 = 15%; (scale bar = 100 μm, x200). The model predicted that the lesion possibly has ALNM (p = 0.83). MS is Lumina B. ABUS = Automated breast ultrasound, ALNM = axillary lymph node metastasis, MD = maximum diameter, MS = molecular subtype.

**Additional file 2:** **Table S1.** Relationship between Ki-67 status and ABUS features.

## Data Availability

All data are true and valid. Any additional information and data are available upon reasonable request and Qiucheng Wang (email: haerbincss@126.com) should be contacted to access the data.

## Abbreviations

BMI: Body mass index
IDC: Invasive ductal carcinoma
MS: Molecular subtype
TN: Triple negative
ABUS: Automated breast ultrasound
ALN: Axillary lymph node
EBC: Early-stage breast cancer
ALNM: Axillary lymph node metastasis
NALNM: No axillary lymph node metastasis
MD: Maximum diameter
ROC: Receiver operating characteristic
AUC: Area under the receiver operating characteristic (ROC) curve
PPV: Positive predictive value
NPV: Negative predictive value
SLNB: Sentinel lymph node biopsy
ER: Estrogen-receptor
PR: Progesterone-receptor
HER-2: Human epidermal growth factor receptor 2
3D: Three-dimensional
FISH: Fluorescent in situ hybridization
