# Supplementary material for: Prediction model of axillary lymph node status using automated breast ultrasound (ABUS) and ki-67 status in early-stage breast cancer
Source: BMC Cancer. 2022 Aug 28;22:929. doi: 10.1186/s12885-022-10034-3 (PMC9420256; doi:10.1186/s12885-022-10034-3)
Supplement: Supplementary file 1 — Additional file 1: Figure S1. (A) ABUS image of 48-year-old woman in stage II (MD = 2.1 cm) breast cancer with ALNM. The coronal plane shows proper nipple position (yellow point). ABUS detected a big hypoechoic lesion (arrows) on outer quadrant in left breast with irregular shape, angular margin, microcalcification, posterior features shadowing, positive hyperechoic halo and retraction phenomenon. (B, C, D, E) ER (+), PR (+), HER2 (1+), Ki-67 = 15%; (scale bar = 100 μm, x200). The model predicted that the lesion possibly has ALNM (p = 0.83). MS is Lumina B. ABUS = Automated breast ultrasound, ALNM = axillary lymph node metastasis, MD = maximum diameter, MS = molecular subtype. [file 12885_2022_10034_MOESM1_ESM.docx]

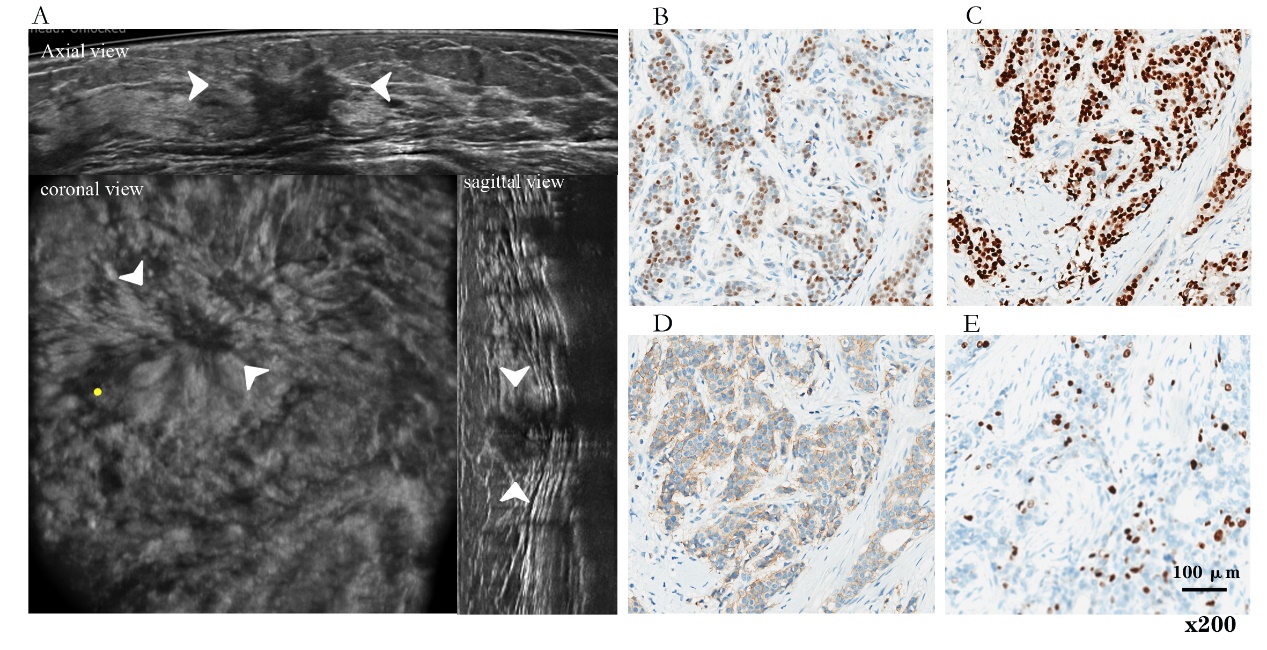


**Figure S1.** (A) ABUS image of 48-year-old woman in stage II (MD = 2.1 cm) breast cancer with ALNM. The coronal plane shows proper nipple position (yellow point). ABUS detected a big hypoechoic lesion (arrows) on outer quadrant in left breast with irregular shape, angular margin, microcalcification, posterior features shadowing, positive hyperechoic halo and retraction phenomenon. (B, C, D, E) ER (+), PR (+), HER2 (1+), Ki-67 = 15%; (scale bar = 100 μm, x200). The model predicted that the lesion possibly has ALNM (p = 0.83). MS is Lumina B. ABUS = Automated breast ultrasound, ALNM = axillary lymph node metastasis, MD = maximum diameter, MS = molecular subtype.
