# Supplementary material for: Prediction model of axillary lymph node status using automated breast ultrasound (ABUS) and ki-67 status in early-stage breast cancer
Source: BMC Cancer. 2022 Aug 28;22:929. doi: 10.1186/s12885-022-10034-3 (PMC9420256; doi:10.1186/s12885-022-10034-3)
Supplement: Supplementary file 2 — Additional file 2: Table S1. Relationship between Ki-67 status and ABUS features. [file 12885_2022_10034_MOESM2_ESM.docx]

**Table S1 Relationship between Ki-67 status and ABUS features**

| Variables | N | Ki-67 (%) median | | q1-q3 | *P* |
| --- | --- | --- | --- | --- | --- |
| Margin |  | |  |  | 0.0244 |
| circumscribed | 51 | | 20 | 10-30 |  |
| spiculated | 240 | | 15 | 10-30 |  |
| angular | 78 | | 20 | 15-40 |  |
| indistinct | 100 | | 20 | 10-30 |  |
| Shape |  | |  |  | 0.0279 |
| regular | 28 | | 10 | 10-22.5 |  |
| irregular | 441 | | 20 | 10-30 |  |
| Echo pattern |  | |  |  | 0.2715 |
| hypoechoic | 434 | | 20 | 10-30 |  |
| heterogeneous / complex cystic and solid | 22 | | 15 | 10-30 |  |
| iso- / hyperechoic | 13 | | 10 | 10-20 |  |
| Calcifications |  | |  |  | 0.0124 |
| no | 213 | | 15 | 10-30 |  |
| micro | 248 | | 20 | 10-35 |  |
| macro | 8 | | 17.5 | 10-25 |  |
| Orientation |  | |  |  | 0.0512 |
| parallel | 431 | | 20 | 10-30 |  |
| not parallel | 38 | | 15 | 10-20 |  |
| Posterior features |  | |  |  | 0.0049 |
| no features | 313 | | 15 | 10-30 |  |
| shadowing | 77 | | 15 | 10-27.5 |  |
| enhancement | 74 | | 30 | 10-50 |  |
| combined pattern | 5 | | 50 | 30-70 |  |
| Hyperechoic halo |  | |  |  | 0.2015 |
| negative | 332 | | 15 | 10-30 |  |
| positive | 137 | | 20 | 10-30 |  |
| Retraction phenomenon |  | |  |  | <0.001 |
| negative | 30 | | 20 | 10-40 |  |
| positive | 160 | | 15 | 10-20 |  |

ABUS = Automated breast ultrasound
